# Novel Airflow-Field-Driven Melt Spinning 3D Printing of Tubular Scaffolds Based on Polycaprolactone Blends

**DOI:** 10.3390/polym15071755

**Published:** 2023-03-31

**Authors:** Junyuan Zhang, Zilong Peng, Mengjie Wang, Yinan Li, Jinyin Wu, Yifan Jiang, Chaolong Liu, Guqiang Li, Lin Xu, Hongbo Lan

**Affiliations:** 1 Shandong Engineering Research Center for Additive Manufacturing, Qingdao University of Technology, Qingdao 266520, China; 2 Key Laboratory of Additive Manufacturing and Applications in Universities of Shandong, Qingdao University of Technology, Qingdao 266520, China; 3 Institute of Rehabilitation Engineering, Binzhou Medical University, Yantai 264003, China; 4 Yantai Affiliated Hospital, Binzhou Medical University, Yantai 264100, China

**Keywords:** airflow-field-driven, 3D printing, microfiber scaffold, polycaprolactone (PCL), polylactic acid (PLA)

## Abstract

The fabrication of various 3D tissue engineering tubular scaffolds with fibrous structures, to assist the human body in rapidly repairing a variety of ailments, is receiving more and more attention. Due to the inefficiency of the majority of fibrous preparation techniques, the question of how to rapidly produce the requisite three-dimensional tubular microfiber scaffold structures has become an urgent problem. In this study, an efficient polymer fiber preparation method was developed, using a high-speed airflow drive. Melt blending of polycaprolactone (PCL), polylactic acid (PLA), and tributyl citrate (TBC), was used for the printing material, to achieve the efficient preparation of tubular microfiber scaffolds with different structures. The scaffold diameter was as small as 2 mm, the wall thickness was up to 100 μm, and the fiber injection efficiency reached 15.48 g/h. By utilizing simulations to optimize the printing parameters and by adjusting the printing settings, it was possible to achieve a controlled fiber diameter in the range of 3 μm to 15 μm. In addition, plasma treatment was applied to the microfibers’ surface, to increase their wettability, and the efficiency of the hydrophilic modification was demonstrated. Furthermore, the mechanical property test demonstrated that the fibers have a tensile strength of 1.36 ± 0.16 MPa and a tensile strain of 30.8 ± 3.5%. The radial compressive strain of the tubular scaffold could reach 60% of the original scaffold’s diameter. Finally, the in vitro degradation of the fibers at various pH values was tested. The results showed that, under alkaline conditions, the surface of the fibers would be severely crushed and the rate of deterioration would increase.

## 1. Introduction

The human body encounters various types of reparable injuries in daily life, some of which might have severe implications if left untreated. To overcome these issues, researchers have created various tubular scaffolds to rapidly repair human injuries. Since the development of biological scaffolds, the employment of efficient ways to produce scaffolds and enhance their structure and performance for clinical applications, has been a hot topic among researchers.

Microfibers have attracted the interest of many researchers, due to their high specific surface area and porosity, which are compatible with the bio-permeability required for biological scaffolds [1]. However, the most prevalent solution, the electrospinning technique, for the preparation of microfibers, suffers from inefficiency and solvent residues, and it typically takes hours or even tens of hours to fabricate fiber structures hundreds of microns thick [2], so more efficient methods are required. Researchers have approached this issue in a variety of ways. For example, Wei et al. [3] created a linear flume spinneret to fabricate polyacrylonitrile (PAN) fibers. This could produce multiple jets and increased fiber productivity to 4.85 g/h. However, this method is not flexible and makes it difficult to obtain small-sized fiber structures, which is also a disadvantage of most multi-jet jets. Vaquette et al. [4] used an electrostatic lens to concentrate the PCL jet onto a smaller area, so as to obtain a thicker fiber structure. The injection efficiency of this method was 0.285 g/h, which is not significantly improved, and its experimental equipment was complex required the use of a high-voltage power supply.

In recent years, advances in the fields of medicine and materials have paved the way for the optimization of biological scaffolds, and the options of materials determine the properties of biological scaffolds, such as the hydrophilic and mechanical properties. Among them, the synthetic polymer materials PLA and PCL, have been approved by the Food and Drug Administration (FDA). They have good biocompatibility and biodegradability, and their degradation products are non-hazardous carbon dioxide and water [5], so they are widely used in the fabrication of scaffolds. Furthermore, the different viscosity of the two materials will affect the printing process, for example, when the material is too sticky, it will hinder the printing process. PLA is more brittle, in contrast, PCL has superior ductility and structural stability [6]. Therefore, mixing different materials is an effective method to solve the defects of a single material [7]. Yu et al. [8], for example, used electrospinning to fabricate nanofibers with 72.9 ± 1.89% porosity, by combining PLA and polyglycolic acid (PGA). Due to the faster degradation of PGA, five layers of neural catheter scaffolds, with varied PGA and PLA ratios, were fabricated, so as to control the sequential degradation of scaffolds. Aminatun et al. [9] used the electrospinning technique to manufacture biodegradable anterior cruciate ligament (ACL) nanofiber scaffolds from a combination of PCL and PLA, demonstrating its excellent mechanical characteristics. The cellular experiment showed a living cell percentage of 97.416 ± 5.079. Since most of the solution spinning methods use toxic solvents, and the residue of toxic solvents will limit the application of fibers in the direction of biomedicine, melt spinning is commonly utilized to prepare microfibers, which can solve the problem of solvent residue and improve fiber preparation efficiency. 

This study proposes a device that can efficiently produce microfibers, by employing a high-speed airflow-driven molten polymer spinning technique. Using a melt blend of PCL/PLA and TBC as the printing material, with TBC serving as a bulking agent for PCL/PLA. The law of the influence of printing parameters on fibers’ morphology was studied, and it was shown that the productivity of fibers reached 15.48 g/h, which is 1–2 orders of magnitude greater than electrospinning. It is worth noting, that this technology has the features of achieving controllable macroscopic routes, and also realizing controllable fiber diameter and porosity within a given range, by modifying printing parameters. Using the effective preparation efficiency of this technology, tubular microfiber scaffolds with diameters of 2 mm, 4 mm, 6 mm, and 8 mm were rapidly prepared, and the smallest wall thickness of the scaffolds was 100 μm. In addition, the surface wettability of the fibers could be altered through plasma treatment. After testing the mechanical properties of the fiber samples, the microfiber scaffolds’ superior mechanical properties are demonstrated. Finally, the fibers’ degradation properties at different pH values were investigated, and the results showed that the fiber diameter decreased the most in a neutral environment, but the fibers appeared to be heavily crushed in an alkaline environment. This study provides some reference ideas and methods for the rapid preparation of tubular microfiber scaffolds.

## 2. Materials and Methods

### 2.1. Materials

Polycaprolactone (PCL) (Mn 80.000) and polylactic acid (PLA) (Mn 60.000), were purchased from Sigma Aldrich (Shanghai, China). The materials were dried in a drying oven at 50 °C for 6 h, before use. Tributyl citrate (TBC, 98% purity) was purchased from Shanghai Aladdin Bio-Chem Technology Co., Ltd. (Shanghai, China). NaOH (particles, 97%), phosphate buffer (PBS, pH7.4), and phosphate solution (AR, ≥85 wt.%), were provided by Shanghai Macklin Biochemical Technology Co., Ltd. (Shanghai, China).

### 2.2. Preparation of PCL/PLA/TBC Fibers

**Figure 1:**
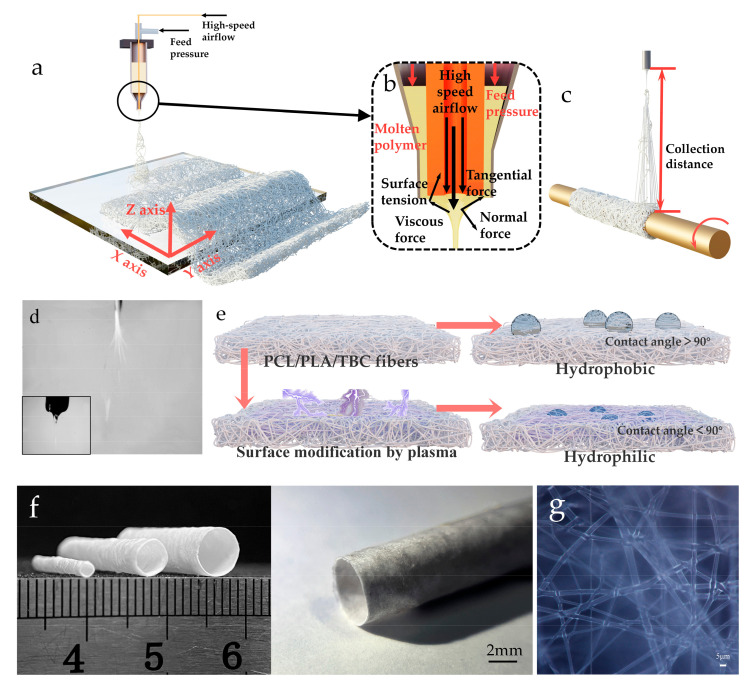
(**a**) Schematic diagram of the 3D printing fiber process. (**b**) Schematic diagram of fiber printing. (**c**) Schematic diagram of the tubular microfiber scaffold printing process. (**d**) The state of the jet stream taken by a high-speed camera. (**e**) Schematic diagram of plasma fiber surface modification process. (**f**) Diagram of finished tubular microfiber scaffolds with different structures. (**g**) Microscopic morphology of the fibers.

In this article, individual fibers are denoted by “fiber” and fiber scaffolds are denoted by “tubular microfiber scaffold”. Figure 1 depicts the methods employed in this work. To make fibers with increased toughness, PCL and PLA were blended in a 7:3 ratio, then TBC was added at an 8% mass ratio for melt blending. The blended material was fed into the barrel, and the barrel and nozzle temperatures of the heating block were adjusted to 160 °C and 180 °C, respectively, for a period of time, in order to thoroughly melt the material.

Figure 1b depicts the internal structure of the nozzle. A 1 mm diameter hollow copper tube is fixed inside the nozzle. The gas flowing into the hollow copper tube is defined as the high-velocity airflow and the gas flowing into the barrel is defined as the feed pressure. Increasing the feed pressure causes the molten material to flow slowly from the nozzle. When the high-speed airflow was passed, shear force stretches the material into microfibers. In addition, when building the tubular microfiber scaffolds, a rotating shaft needed to be installed under the nozzle and the fibers were collected using the three-axis drive system and the tubular structure on the rotating shaft [10], as shown in Figure 1c. Figure 1d depicts the fiber jet flow, as captured by a high-speed camera, and Figure 1e depicts a schematic diagram of the hydrophilic modification of the plasma-treated fibers’ surface. Figure 1f,g show the printed catheter microfiber scaffolds with different structures, and the microscopic morphology of the fibers.

### 2.3. Microscopic Morphological Characterization

The surface morphology of the PCL-PLA-TBC fibers was characterized using a field emission scanning electron microscope (Merlin-Compact, Carl-Zeiss, Oberkochen, Germany), and a optical microscope (DSX510, Olympus, Tokyo, Japan). The fibers’ images were observed using the ImageJ software, and 50 fiber diameters were randomly selected from each image for measurement. The data whose difference from the mean was greater than three standard deviations, were deleted during processing. The porosity of the fibers was measured using the queue segmentation method [9].

### 2.4. Fourier-Transform Infrared Spectrophotometer Spectra

The spectral relationship between transmittance and wave number (cm^−1^) was evaluated by analyzing the functional groups of the samples with FTIR (Invenio, Bruker, Saarbrucken, German), in the wave number range 400–4000 cm^−1^, to determine the chemical properties and elemental interactions of PCL, PLA, TBC, and the mixed materials.

### 2.5. Wetting Performance and Mechanical Properties Testing

Hydrophilic modification of the fibers’ surface using plasma treatment and hydrophilic modification before and after the surface contact angle of water droplets on the surface of the fiber samples, were measured with contact angle measuring instrument (JC2000D, Zhongchen, Shanghai, China), and all surface contact angles of the fiber samples were tested over five times.

The mechanical properties of the fiber samples were determined using a mechanical testing machine (CMT6503, SANS, Shenzhen, China). The fiber samples were cut into strips of 50 mm length, 5 mm width, and 200 μm thickness for the tensile test, and the load–deformation curves were recorded. The radial compression test was performed on tubular microfiber scaffolds with a diameter of 4 mm and a wall thickness of 200 μm. The load–deformation curves were recorded when the support was radially compressed until the tubular microfiber scaffolds collapsed. Each sample was tested three times for tensile or compressive limits. The tensile or compressive strength of the samples can be calculated according to the classical stress formula [9].

### 2.6. In Vitro Degradation

PBS was used as a buffer, and the hydrophilic treated fiber samples were placed in Petri dishes at pH 3, 7.4, and 10, respectively, and placed in an incubator with a shaker at 37 °C, to simulate physiological conditions. The Petri dishes were removed every three days and the PBS buffer was replaced. After 30 days, the fiber samples were removed and dried in a drying oven at 40 °C for 6 h. The fibers’ microforms were characterized using electron microscopy after gold spraying.

## 3. Results and Discussions

### 3.1. Fourier-Transform Infrared Spectroscopy

**Figure 2:**
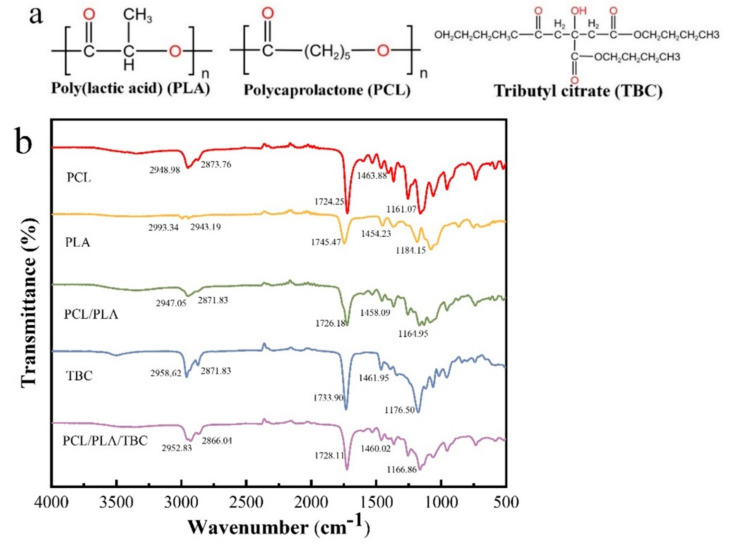
(**a**) Chemical formulae of the main materials. (**b**) FTIR spectra of samples with the composition of PCL, PLA, PCL/PLA, TBC, and PCL/PLA/TBC.

FTIR analysis was used to detect possible changes in the chemical structure of the materials after mixing. Figure 2a shows the chemical formulae of the main materials. Figure 2b depicts the FTIR spectra of PCL, PLA, PCL/PLA, TBC, and PCL/PLA/TBC.

As can be seen from Figure 2, in the FTIR spectrum of the pure PCL sample, the CH_2_ stretching vibration absorption peaks are at 2948 cm^−1^ and 2873 cm^−1^, the CH_2_ bending vibration absorption peaks are at 1463 cm^−1^ and 1150–1350 cm^−1^, the C=O stretching vibration absorption peak is at 1724 cm^−1^, and the C–O stretching vibration peak is between 1000 cm^−1^ and 1300 cm^−1^, indicating the presence of ester groups in the molecule. In addition, a small absorption peak can be observed at 3510 cm^−1^, which is related to the O–H bond of the PCL end group [11,12]. In the IR spectra of pure PLA samples, C–H stretching vibrational peaks were observed at 2993 cm^−1^ and 2943 cm^−1^, CH_3_ bending vibrational peaks at 1454 cm^−1^ and 1371 cm^−1^, C=O stretching vibrational peaks at 1745 cm^−1^, and C–O stretching vibrational peaks between 1000 cm^−1^ and 1300 cm^−1^. In the PCL/PLA blend, these peaks appear in the corresponding neutralization zone [13].

In the FTIR spectrum of the TBC sample, an O–H absorption peak was observed at 3510 cm^−1^, which indicates the presence of O–H functional groups. Similarly, 2958cm^−1^ and 2871 cm^−1^ are C–H stretching vibrations, and 1733 cm^−1^, 1176 cm^−1^, and 1064 cm^−1^ are also observed absorption peaks, which indicate the presence of ester groups in the molecule. The absorption peaks were also observed at 1456 cm^−1^ and 1386 cm^−1^, indicating the presence of methyl structures in the molecule, which is consistent with the molecular structure of TBC. The absorption peaks of the PCL/PLA/TBC blends were also in the corresponding neutralization regions, indicating that the blends were well formed.

### 3.2. Airflow Field Simulation

**Figure 3:**
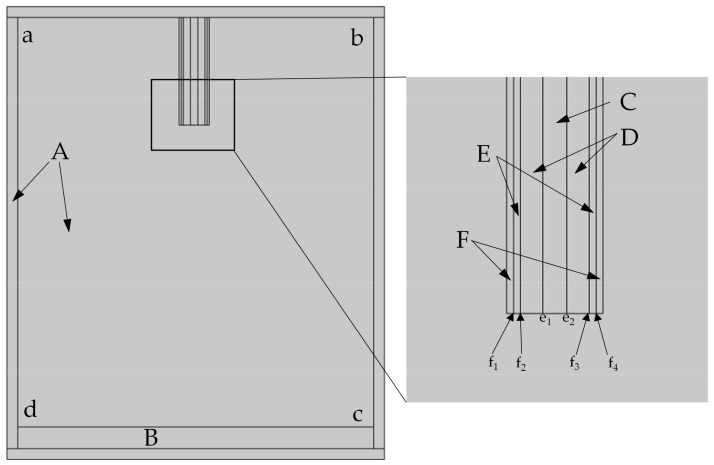
Simulation model.

Simulations can help with printing parameter optimization, and the flow distribution and diffusion range of high-speed airflow at different flow rates were studied using COMSOL finite element simulation. From this, the printing resolution under different printing parameters was obtained. The model was simplified to improve calculation performance, and a two-dimensional model was used for the simulation. Figure 3 shows the established COMSOL simulation model, while Table 1 gives the materials in different parts of the model [14]. Where A represents the surrounding air, B represents the glass substrate, C represents the hollow part of the hollow copper tube, D represents the solid part of the hollow copper tube, E is the gap between the nozzle and the hollow copper tube, and F is the outer wall of the nozzle.

The boundary conditions added in the model are shown in Table 2. Airflow velocities of 60, 90, 120, 150, 180, and 210 m/s are applied to e_1_–e_2_ in the hollow copper tube, and an airflow velocity of 20 m/s is applied to f_1_–f_2_ and f_3_–f_4_ at the material outflow, below the model nozzle, according to the material outflow at 30 kPa under actual conditions.

The speed of the high-speed airflow velocity attenuation, and the size of the diffusion range, can be calculated. This is the fiber jet simulation range.

**Figure 4:**
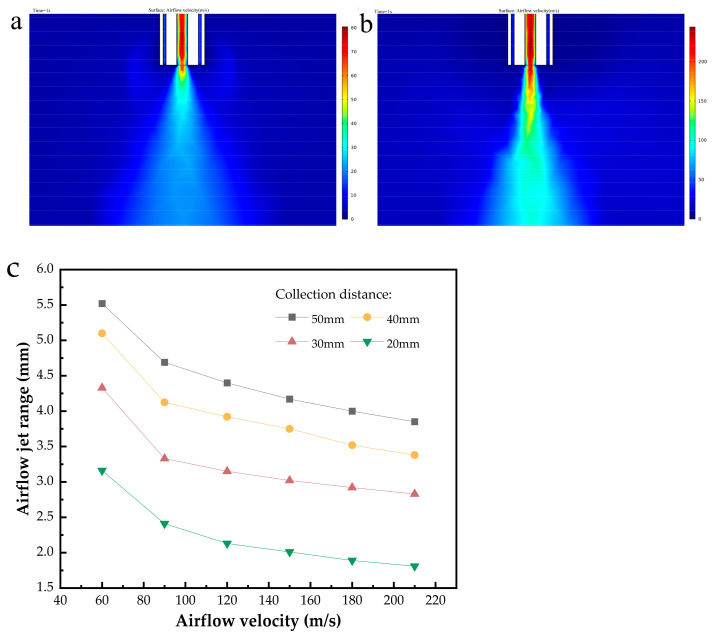
(**a**,**b**) Simulation diagram when the high-speed airflow is 60 m/s and 210 m/s, respectively. (**c**) Airflow diffusion range under different parameters.

The purpose of modeling is to simulate the airflow diffusion range in order to obtain the fiber jet range, which aids in parameter selection during printing. Figure 4a,b show the modeling results with high-speed airflows of 60 m/s and 210 m/s, respectively. The diffusion range of the high-speed airflow was measured at different collection distances, and the results are shown in Figure 4c. As can be observed, as the velocity of high-speed airflow increases, the airflow becomes much more focused, and the diffusion of the airflow decreases. Furthermore, when the collection distance decreases, so does the diffusion range of the jet stream. This can help in the optimization of fiber printing parameters, particularly when printing tubular microfiber scaffolds with small diameters. To ensure that the fiber diffusion range is as small as possible, a larger airflow velocity and smaller collection distance should be chosen as far as feasible in the real situation. For example, when printing a tubular microfiber scaffold with a diameter of 4 mm, we selected a 180 m/s high-speed airflow and 40 mm collection distance, according to the simulation, and the subsequent printing results verified the reliability of the simulation.

### 3.3. Fibers’ Morphology Characterization of PCL/PLA/TBC

**Figure 5:**
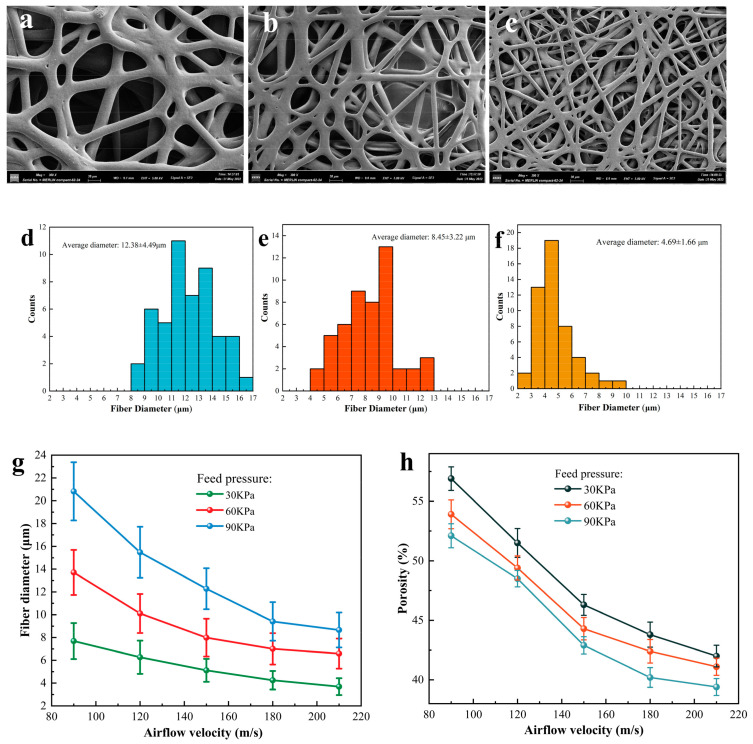
(**a**–**c**) Microscopic images of fibers corresponding to different printing parameters, at the same magnification. (**d**–**f**) Statistical diagram of fiber diameter. (**g**) Relationship between printing parameters and fiber diameter. (**h**) Relationship between printing parameters and fiber porosity.

The parameters of the printing process, such as feed pressure and high-speed airflow, can have a considerable effect on the fibers’ microscopic morphology, therefore, printing parameter adjustments can be investigated, to regulate the fibers’ microscopic morphology. The collection distance was set to 40 mm, and the high-speed airflow was kept at 150 m/s, while the feed pressure was adjusted to 30 kPa, 60 kPa, and 90 kPa. The resulting fibers were observed using a scanning electron microscope (SEM), with the same magnification, and the results are shown in Figure 5a–c. Figure 5d–f shows the corresponding statistical diagram of fiber diameter. To determine the significance of the influence of the process parameters on the fiber diameter, the two-sample *t*-test was used for data collected under different process parameters, and the *p*-value was calculated. When the *p*-value was less than 0.05, it indicated that there was a statistically significant difference between the two data groups. The results indicate that when the feed pressure is 30 kPa and the high-velocity airflow is 180 m/s and 210 m/s, the *p*-value of the two data groups is the largest, which is 0.0334. This indicates that the process parameters have a great influence on the fiber diameter. Furthermore, it is obvious from Figure 5a–c, that the fiber diameter increases with the increase in the feed pressure, which is due to the higher feed pressure increasing the amount of material flowing out of the nozzle per unit of time, and the high-speed airflow is not enough to shear and refine the material, so the fiber diameter becomes thicker. When the feed pressure remains constant, the fiber diameter decreases with increasing high-speed airflow, as seen in Figure 5g. In addition, it should be noted that, in order to ensure the continuity of the printing process, we cannot simply reduce the feed pressure and increase the high-speed airflow to get a finer fiber structure, so the high-speed airflow is selected to be around 150 m/s, and the feed pressure is generally selected as 30 kPa. In addition, with the increase in high-speed airflow, the average diameter of the fiber will tend to a limit, with the finest diameter being roughly 3.5 μm.

The porosity of the fiber was measured using threshold segmentation of SEM images, where the dark color represented the background and the light color indicated the fiber. Using the Image J software, Version 1.53t, we performed the area fraction analysis, and the area ratio of the dark area was the porosity value. Figure 5h shows the porosity of the fibers under different printing parameters. As previously, it is calculated that when the high-speed airflow is 120 m/s and the feed pressure is 60 kPa and 90 kPa, the *p*-value of the two groups of data is the largest, which is 0.0445. This proves that changing the parameters has an obvious effect on the fiber porosity. It can be seen from Figure 5, that when the flow rate of the high-speed airflow is kept constant, the porosity of the fibers reduces with increasing feed pressure, which is also influenced by increasing the flow rate of the molten material out of the nozzle per unit time, making the fibers denser. When the feed pressure remains constant, the porosity of the fibers decreases as the velocity of the high-speed airflow increases. According to the simulation results, increasing the high-speed airflow leads to a decrease in the diffusion area of the airflow, and the obtained fiber range is smaller, which means that the same amount of fibers fall in a smaller range. Simultaneously, the higher speed airflow’s impact will compact the fibers more tightly, resulting in a decrease in porosity. In summary, the diameter and porosity of fibers can be controlled, within a certain range, using printing parameters.

**Figure 6:**
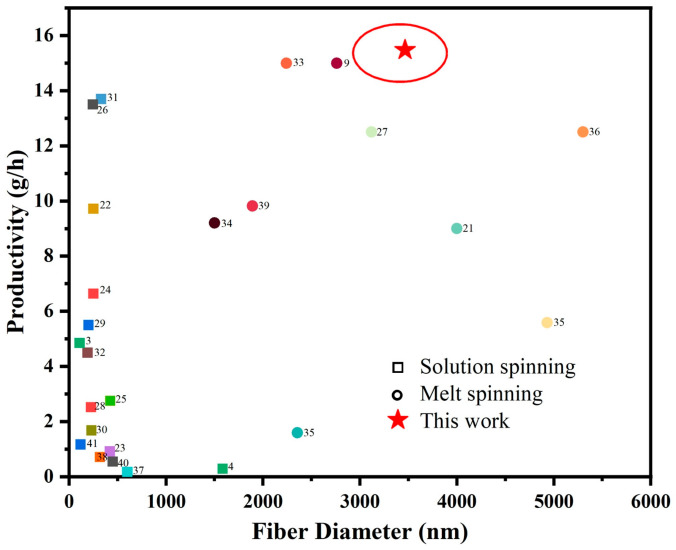
Comparison graph of fiber injection efficiency for different methods [3,4,15,16,17,18,19,20,21,22,23,24,25,26,27,28,29,30,31,32,33,34,35].

Figure 6 shows a comparison of the efficiency and fiber diameter, of fiber preparation methods that have been reported in recent years. Where squares represent the solution spinning technique and circles represent the melt spinning technique. It can be seen that the method presented in this paper, has a significant advantage in fiber preparation efficiency, reaching up to 15.48 g/h. However, the fiber diameter obtained by the solution spinning method is usually in the nanometer scale, whereas the fiber diameter obtained by the melt spinning technique is usually in the micron scale, and the average diameter of the fibers prepared by the method used in this paper is about 3.5 μm. As a result, the following step, such as the intervention of an electric field to further refine the fiber diameter, in order to produce a higher specific surface area and more biometric results, should be considered.

### 3.4. Tubular Microfiber Scaffolds and Characterization of Their Properties

**Figure 7:**
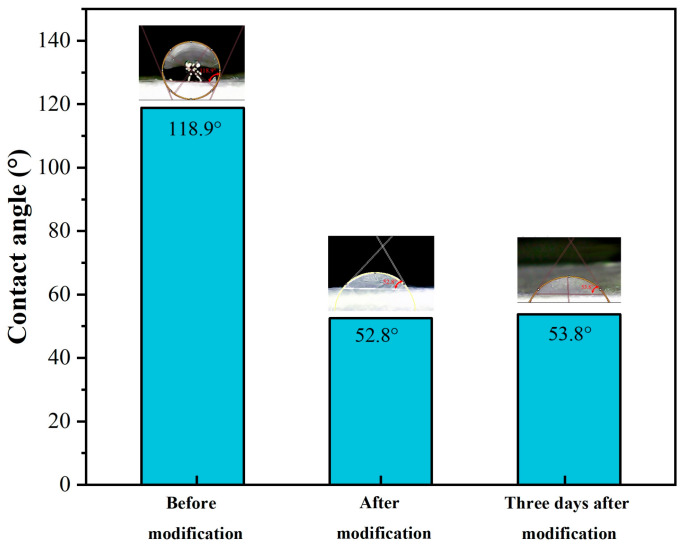
The surface contact angle of fiber samples before and after hydrophilic modification.

Wettability is an important measure of a biological scaffold, and excellent hydrophilic properties can facilitate nutrient exchange inside and outside the tubular microfiber scaffold. The printed fiber samples will display hydrophobic properties due to the hydrophobic groups of the PCL and PLA materials, with a measured contact angle of 118.9°, as illustrated in Figure 7. Cold plasma treatment is a promising strategy, since it can improve a material’s surface adhesion and wettability without changing its elastic modulus [7]. After using plasma treatment on the fiber sample’s surface for 5 min, we measured the surface contact angle of the fiber sample as 52.8°, indicating that the fibers exhibited good hydrophilicity after plasma treatment. Then, the fiber samples were stored at 0–4 °C for three days before remeasuring the contact angle, and there was no significant change in the contact angle, as shown in Figure 7. This shows that the hydrophilic modification method is effective, and that the hydrophilic nature can be maintained for a long period of time.

**Figure 8:**
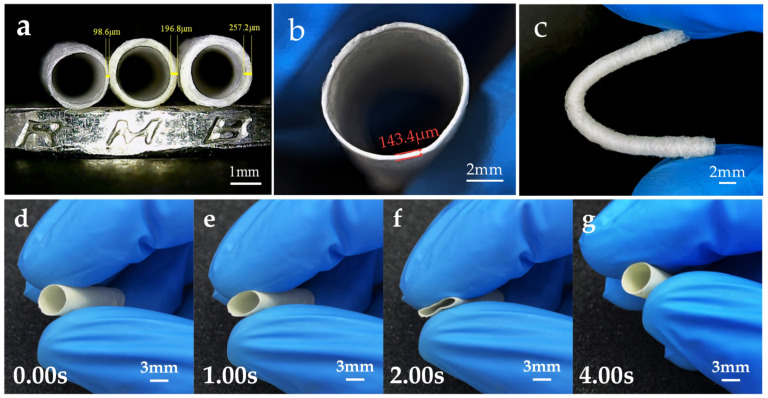

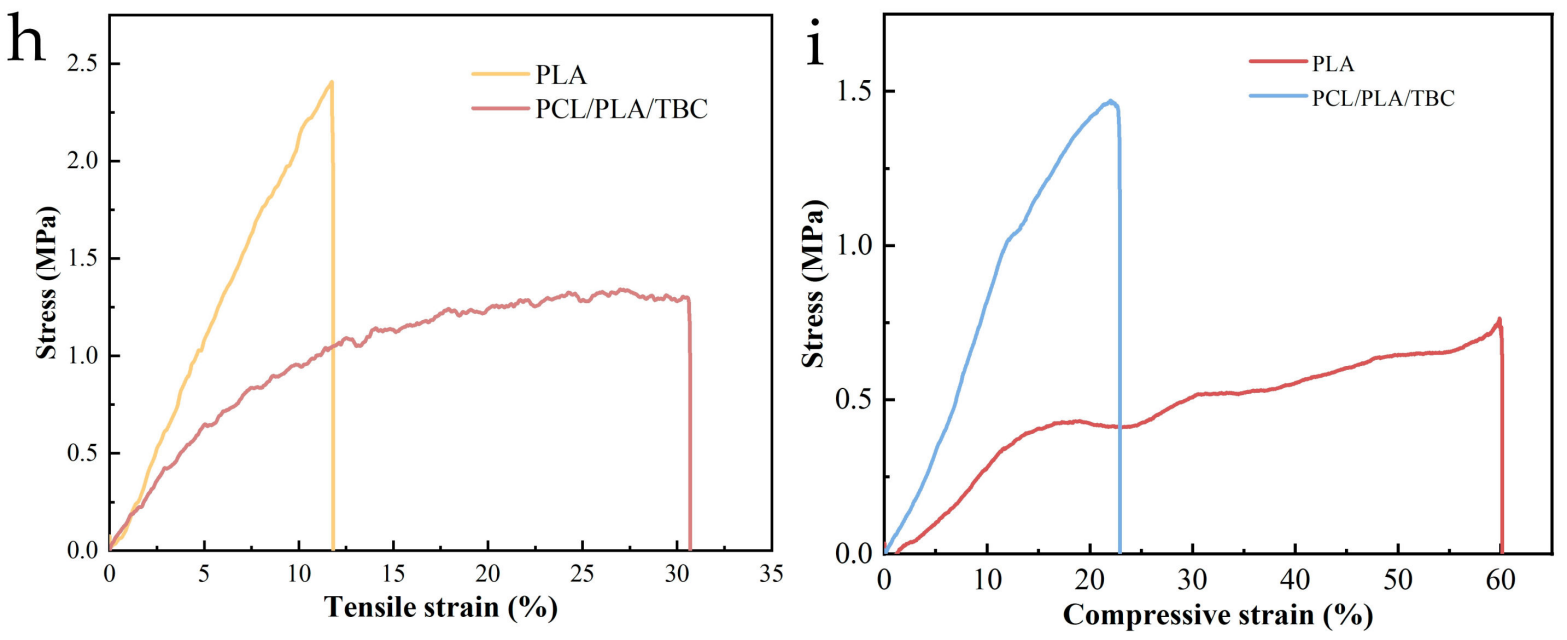
(**a**–**c**) The tubular microfiber scaffolds with different structures. (**d**–**g**) Compressive resilience of tubular microfiber scaffolds. (**h**) Tensile stress–strain of different fiber samples. (**i**) Axial compressive stress–strain of different tubular microfiber scaffolds.

The optimized printing parameters obtained from the simulation and experiment were used to print the tubular microfiber scaffold on the rotational axis, and the printed tubular microfiber scaffolds are shown in Figure 8a–c. The minimum diameter of the tubular microfiber scaffolds produced is 2 mm, and the finest wall thickness can be controlled to about 100 μm. Notably, relying on the efficient preparation efficiency of this method, it takes only ten seconds to print one of the microfiber scaffolds shown in the figure. Figure 8d–g demonstrate the good resilience of the tubular microfiber scaffolds after radial compression.

The mechanical properties are also significant in evaluating bio-scaffolds. The printing effect is poor, due to the high viscosity of the PCL. PLA and PCL/PLA/TBC materials were chosen to print fiber samples for tensile and compressive performance testing and comparison. Figure 8h depicts the fibers’ tensile stress–strain graph. The tensile strength of PLA is higher, at 2.41 ± 0.22 MPa, but its tensile strain is only 11.7 ± 1.2%, whereas the tensile strain of fibers made from the PCL/PLA/TBC material is substantially larger, reaching 30.8 ± 3.5%. Similarly, as illustrated in Figure 8i, the compressive properties of the tubular microfiber scaffolds were investigated. As for the tensile qualities, the scaffolds printed with pure PLA materials could only achieve a compressive strain of about 22%, and their overly brittle characteristics prevent them from being more widely used. In contrast, the co-blended material tubular microfiber scaffolds had significantly enhanced compressive strains, up to 60.2 ± 4.6%, making this material more suited for tubular microfiber scaffold production.

### 3.5. In Vitro Degradation Experiments

PCL and PLA have rather lengthy biodegradation times, with PLA typically degrading in 10 months to 4 years and PCL typically degrading in 2 years [9]. Biological scaffolds that cannot be degraded for a long time may cause thrombosis and other dangers. To reduce the degradation cycle of fibers, the fiber degradation under different pH values was studied.

**Figure 9:**
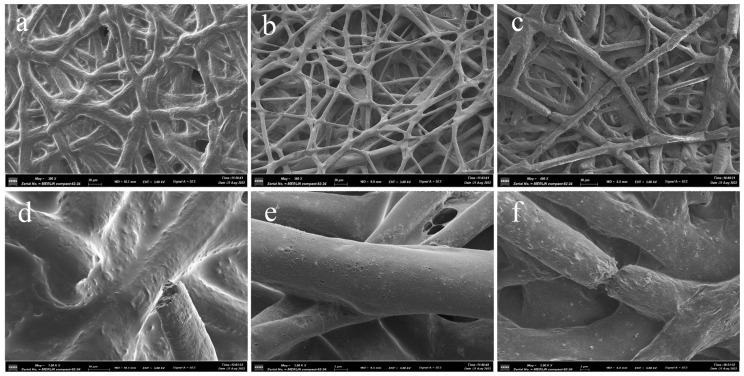
(**a**–**c**) SEM images of the degraded PCL/PLA/TBC fibers at pH values of 3, 7.4 and 10 respectively. (**d**–**f**) SEM images of the degraded single fiber at pH values of 3, 7.4 and 10 respectively.

Phosphoric acid solution and NaOH particles were added to PBS buffer, to adjust the pH to 3 and 10, respectively. Fiber samples of 40 × 10 mm, were placed in the PBS buffer with pH values of 3, 7.4, and 10, for in vitro degradation tests, and the PBS buffer with different pH values was changed every three days during the test period. After 30 days, the degradation of the fiber samples was detected. The macroscopic surface of the fiber samples in the alkaline environment was observed to be noticeably crushed, whereas the appearance of the remaining two sets of fiber samples did not change considerably. Furthermore, their surface microscopic morphology was studied separately. Figure 9a–c illustrate the microscopic morphology of the fibers at pH 3, 7.4, and 10, respectively, while Figure 9d–f depict the local microscopic pictures of a single fiber in the appropriate environment. Table 3 shows the diameter variations before and after fiber degradation at different pH values. The fiber diameter is shown to drop slightly following degradation in an alkaline environment, and there are many fractures. In the acidic environment, the fiber’s surface is corroded by acid, causing adhesion between fibers, and the fiber diameter is distorted and thickened due to corrosion, with a few cracks. In the neutral atmosphere, the surface of the fibers revealed dents, and the average diameter of the fibers dropped by roughly 0.8 μm, compared to before degradation. 

When compared to fiber degradation in the neutral environment, the macro and microscopic morphology, as well as mechanical properties, of fibers in the alkaline environment change significantly, and the fibers are crushed within 30 days, which is primarily due to the fact that the majority of the mixed material is PCL, and PCL degradability is faster in an alkaline environment [36]. Because of the corrosive effects, the fibers in an acidic environment will cling together, which will have a higher influence on the porosity of the fibers and is not suited for fast degradation.

## 4. Conclusions

This paper presents an efficient fiber preparation method for high-velocity airflow-driven molten polymers, which primarily utilizes the shear force of high-velocity airflow on molten materials to stretch and refine them into fibers, with the benefits of high efficiency, without the need for a solvent or a high-voltage power supply. Biocompatible PCL and PLA were used as the printing materials, and TBC was used as the bulking agent for melt blending. The effect of the printing parameters on the fibers’ morphology was studied, the normal printing efficiency can reach 15.48 g/h, and control of the fibers’ diameter, by varying the printing parameters, was achieved, with the smallest average fiber diameter measuring approximately 3.5 μm. In addition, rapid molding of 3D tubular microfiber scaffolds, with various structures, can be produced, by utilizing the effective fiber preparation efficiency of this technology. Furthermore, the tensile strength of the fiber samples was 1.36 ± 0.16 MPa and the tensile strain was 30.8 ± 3.5%, while the radial compressive strain of the tubular microfiber scaffolds was 60.2 ± 4.6%, and the tubular microfiber scaffolds have good resilience after radial compression. The plasma treatment for surface modification was applied to the fibers, to improve their wettability. Finally, the printed fibers were subjected to in vitro degradation at different pH values, and the results showed that, the alkaline environment had the greatest effect on the degradation of the fibers prepared from this material. This work introduces a novel method for printing and surface modification of tubular stents, with excellent efficiency.

## Figures and Tables

**Table 1:** Simulation model zones and their material properties.

	Regional Division	Material
A	Infinite metadomain	Air
B	Substrate	Glass
C	Vacant position	Air
D	Hollow copper pipe wall	Copper
E	Printed material	PCL
F	Nozzle	Copper

**Table 2:** Boundary conditions.

Position	Boundary Condition
e_1_–e_2_	Normal inflow velocity = 60, 90, 120, 150, 180, and 210 m/s
f_1_–f_2_, f_3_–f_4_	Normal inflow velocity = 20 m/s

**Table 3:** Changes in average fiber diameter at different pH values.

Fiber Samples	pH	Fiber Diameter (μm)	
		Before Degradation Test	After Degradation Test
Sample A	3	5.52 ± 1.42	8.58 ± 1.67
Sample B	7.4	5.31 ± 1.31	4.51 ± 1.04
Sample C	10	5.36 ± 1.37	5.09 ± 1.29

## Data Availability

Not applicable.
